# Nationwide screening program for early detection of oral mucosal cancer - A review

**DOI:** 10.6026/973206300212429

**Published:** 2025-08-31

**Authors:** Kriti Shrivastava

**Affiliations:** 1 Department of Oral Medicine and Radiology, Rishiraj College of Dental Sciences & Research Centre, Bhopal, Madhya Pradesh, India

**Keywords:** Oral cancer, screening program, early detection

## Abstract

India accounts for a significant global burden of oral cancer, despite this; the country lacks an exclusive national oral mucosal cancer screening program. Current efforts are limited to screening under broader non-communicable disease programs. Establishing a national Program integrated within existing health infrastructure could be a transformative step in cancer prevention. Therefore, it is of interest to focus on the urgent implementation of a targeted screening initiative to improve early diagnosis and reduce the burden of oral cancer in India.

## Background:

Oral cancer (Oral Squamous Cell Carcinoma) is a major public health issue in India (both in rural and urban regions) and increasing usage of tobacco (smoked and non-smoked) is one of the potential causes for its soaring prevalence [1]. Despite its avertable nature, it continues to claim a huge number of lives each year [2]. With the highest global burden of oral cancer cases, India (followed by China and US), stands at a crucial choice: either continue to treat this disease at advanced stages with poor survival rates or take appropriate measures for early detection and prevention through a well-structured and planned national oral mucosal cancer screening program [3]. Therefore, it is of interest to implement a nationwide screening program for early detection of oral mucosal cancer.

## Prevalence and impact:

As per epidemiological statistics India accounts for nearly one-third of the global oral cancer burden, with approximately 77, 000 new cases and more than 50,000 deaths per year. The 5 year survival rate is less than 50% due to late stage diagnosis [4]. Among Indian men (especially in the 40-60 years, although the cases are increasing in young individuals also ), it ranks among the top three cancers, largely due to the widespread use of smoked and smokeless tobacco, betel nut and alcohol-especially in lower-income and rural populations. While most oral cancers are often preceded by visible potentially malignant lesions and conditions -such as leukoplakia, Oral Submucous Fibrosis etc-these early are frequently missed due to lack of awareness, absence of routine screening and inadequate training of primary healthcare providers in oral mucosal examination [5].

## What's missing in our screening and early detection approach?

National Oral Health Program runs in the country but it does not focus exclusively on cancer. There are certain state level initiatives for screening of oral cancer in states like Tamil Nadu and Kerala, also AIIMS and certain dental colleges and NGO's are also locally involved in oral cancer screening programs, but currently there is no National oral mucosal cancer screening program in India. Programs such as the National Programme for Prevention and Control of Cancer, Diabetes, Cardiovascular Diseases and Stroke (NPCDCS) focus mainly on breast, cervical cancer [6].

## Additionally, primary healthcare workers:

Accredited Social Health Activists (ASHAs) and even general dentists and physicians, are not trained enough in intraoral visual inspection techniques. Oral medicine specialist who are best positioned and most qualified to detect early lesions-are often not available in community screening models.

## Justification for screening and inclusion of innovations and technology:

A randomised control trial done in Kerala in 2005 is considered to be a landmark study which concluded that trained health workers performing visual inspection could significantly reduce oral cancer mortality by early detection and prevention [1]. Unlike other cancers that require high end imaging diagnostics, oral cancer can be detected in its early stages with simple diagnostic set: a light source and trained skilled eyes. This visual screening is non-invasive, low-cost and repeatable these advantages make it exceptionally suitable for resource-limited settings [7, 8]. Today's emerging technologies and digital world offers new tools to bridge the screening gap. Various health applications and cloud-based data sharing platforms can significantly improve outreach. These innovations, when incorporated into primary care, can help even non-specialist health workers to participate in early detection. Tele-dentistry can also prove to be a helpful platform especially for underserved areas.

## Recommended interventions:

An efficient national oral mucosal cancer screening program must be launched. The other recommendations are

[1] Oral cancer screening should be formally integrated into NPCDCS with clearly defined guidelines and budget

[2] Training Programs for ASHAs, ANMs and dental auxiliaries in oral mucosal screening.

[3] Public Awareness Campaigns: multilingual awareness programs, skits (nukkad nataks)etc should be organised to educate public

[4] Inclusion of Tobacco Cessation Centres (TCC) at PHC level

[5] Interdisciplinary Approach: dental colleges, medical institutions, NGOs etc can coordinate in this direction to provide early benefits to the patient

## Potential benefits:

In addition to reducing mortality, early detection reduces the financial burden of treatment on patient as well as the government and improves the patient's quality of life. It will also reduce health inequalities, promote general awareness and may also lead to change in cultural attitudes and promote interdisciplinary coordination.

## Conclusion:

India is the epicentre of a preventable and avertable cancer epidemic. A national oral mucosal screening program can prove to be a pivotal health intervention. It is high time for the paradigm shift; the narrative from late-stage treatment should now shift to early diagnosis and prevention. India has the knowledge, infrastructure and workforce to implement a national oral cancer screening program, which should now be put to judicious use.
